# Porridge for influenza: Protocol for a systematic review and meta-analysis

**DOI:** 10.1097/MD.0000000000031473

**Published:** 2022-11-18

**Authors:** Haoxiang Sun, Manxue Mei, Jiayu Xu, Xiaofang Chen, Wei Zhu, Jianping Song

**Affiliations:** a Artemisinin Research Center, Guangzhou University of Chinese Medicine, Guangzhou, China; b The Second Clinical College, Guangzhou University of Chinese Medicine, Guangzhou, China.

**Keywords:** influenza, meta-analysis, porridge, protocol

## Abstract

Influenza is a common disease. If it is not treated in time, it may induce related chronic respiratory diseases and seriously endanger people’s health. Porridge, as a food of the East, has been recorded by doctors of all ages to treat influenza with porridge. However, due to insufficient research, there is a certain controversy about porridge in the treatment of influenza. We therefore plan to conduct a systematic review and meta-analysis to collect data from all published studies on this issue in order to obtain reliable evidence.

**Method and analysis:**

We will search for relevant trials in various databases published by December 2022. To study the efficacy and safety of a RCT of porridge in the treatment of influenza. Standardized data tables will be used to complete data search and extraction in duplicate. All differences will be resolved by consensus. The main result was to observe the symptom score of influenza patients, and the secondary results included body temperature, nasal secretions, nasal resistance and viral culture titers in the nasal secretions. Data synthesis and statistical analysis will be performed for each outcome with Stata V.14.0.

**Results:**

Our study will be a systematic review and meta-analysis to evaluate the efficacy and safety of porridge in the treatment of influenza.

**Conclusion:**

The conclusion of this study has a certain reference value for the clinical use of porridge in the treatment of influenza.

## 1. Introduction

Influenza is a common respiratory disease in clinic. It is an acute respiratory infection caused by ribonucleic acid virus, which has a high incidence in autumn and spring. The main clinical symptoms are fever, cough, runny nose, sneezing, headache, weakness, myalgias or diarrhea, abdominal pain, or vomiting.^[1]^ Influenza is highly contagious and lethal, and its main mode of transmission is through droplets. After infection, the incubation period of the virus is 24 to 48 hours, and it is contagious 1 to 2 days before the onset of symptoms and 2 to 7 days after the onset of symptoms. Between 2010 and 2018, there were about 9.3 million to 49 million cases of seasonal flu in the United States, with 12,000 to 79,000 deaths each year. As many as 500,000 people around the world die of influenza every year, seriously endangering people’s health.^[2]^ The initial stage of the disease is mostly caused by viral infection. It is easy to transform into lower respiratory tract infection and cause other tissue damage, if not treated in time and effectively. At the same time, it may cause the aggravation of chronic respiratory diseases such as asthma, Chronic Obstructive Pulmonary Disease and so on.^[3,4]^ At present, anti-influenza drugs are often used to treat influenza, such as the neuraminidase inhibitors oseltamivir, zanamivir and peramivir, but these drugs have side effects and can cause nausea and vomiting. Some studies have shown that lower temperatures and lower humidity can lead to colds and flu, and can lead to the spread of influenza.^[5,6]^ Some studies have shown that hot chicken soup and hot drink can affect the flow rate of nasal mucus,^[7]^ while higher humidity and higher temperature have a certain therapeutic effect on colds and influenza,^[8–10]^ which shows that hot drinks play a certain role in the treatment of influenza.

Porridge is one of the staple foods on eastern table, which is nutritious and easy to digest. It is suitable for 4 seasons, both young and old. Porridge has been used as a way to treat diseases in China since ancient times.^[11]^ Treatise on febrile Diseases is a work of clinical use of traditional Chinese medicine, which guides the principles of drug use of traditional Chinese medicine practitioners in clinical treatment. It was created by Zhang Zhongjing, a master of traditional Chinese medicine, there are 34 articles about the use of porridge in Treatise on febrile Diseases, of which there are >20 records of exogenous diseases. For example, Article 12 of Treatise on febrile Diseases states: “after taking medicine, you need to take porridge to enhance the efficacy.”^[12]^ Many Chinese people think that chicken soup is too greasy, so they will not choose to use chicken soup when treating diseases. Porridge, as a traditional hot drink in the East, has certain anti-inflammatory and antiviral effects, and the occurrence of influenza is closely related to viral.^[13,14]^ At this time, porridge has become the first choice for the treatment of influenza. Eating porridge may improve nasal secretions, nasal resistance and virus culture titer in nasal secretions of patients with influenza, but there is little comprehensive evidence of randomized controlled trials (RCT) of porridge in the treatment of influenza. Our meta-analysis review aims to obtain reliable estimates of the efficacy and safety of porridge in the treatment of influenza from the data of all relevant RCT.

## 2. Methods

### 2.1. Study design

This protocol is reported in line with the Preferred Reporting Items for Systematic Review and Meta-Analysis Protocols guidelines. This study will be conducted according to the Preferred Reporting Items for Systematic Reviews and Meta-Analyses statements.^[15]^

### 2.2. Study registration

This protocol of systematic review has been registered in the International Prospective Register of Systematic Reviews (PROSPERO). The number is CRD42022313391.

### 2.3. Eligibility criteria

#### 2.3.1. Participants.

All the patients enrolled in the study were adults (over the age of 18) and were diagnosed with influenza. Exclusion criteria: diabetic patients and minors (under 18 years old). The diagnosis of influenza can be based on the diagnosis of influenza in the Influenza diagnosis and treatment Guide (2020 Edition).

#### 2.3.2. Interventions.

We plan to analyze and compare the groups that use porridge to treat influenza with the control group.

Preparation of porridge: 100 g rice, pure water 500 mL, boil for 30 minutes.

#### 2.3.3. Controls.

Treatment as usual or no interventions.

#### 2.3.4. Outcomes.

The main result was to observe the symptom score of cold patients, and the secondary results included body temperature, nasal secretions, nasal resistance and viral culture titers in the nasal secretions.

#### 2.3.5. Study types.

RCTs will be included in our study. Retrospective or prospective observational cohort studies, cross-section studies, case–control studies and reviews will be excluded.

### 2.4. Search strategy

We will conduct a comprehensive and systematic search of the following databases, including PubMed, China National Knowledge Infrastructure, Wanfang, China Science and Technology Journal Database. Two researchers will search all databases mentioned above from 1990 to present. Relevant keywords and their combinations will be applied in the retrieval conditions. Details regarding how the databases will be searched are provided in Table 1.

**Table 1:** Search strategy of PubMed.

Search strategy (PubMed)	
Search	Query
1#	“Influenza” [MeSH Terms]
2#	“Influenza” [Title/Abstract] OR “Human Influenzas” [Title/Abstract] OR “Influenzas, Human” [Title/Abstract] OR “Influenza” [Title/Abstract] OR “Human Flu” [Title/Abstract] OR “Grippe” [Title/Abstract] OR “Influenza in Humans” [Title/Abstract] OR “Influenza in Human” [Title/Abstract] OR “acute respiratory tract infections” [Title/Abstract] OR “cold” [Title/Abstract] OR “upper respiratory tract infection” [Title/Abstract] OR “viral respiratory tract infection”
3#	“ Porridge “ [MeSH Terms]
4#	“Porridge” [Title/Abstract] OR “white rice porridge” [Title/Abstract] OR “the rice gruel” [Title/Abstract] OR “millet porridge” [Title/Abstract] OR “two rice porridge” [Title/Abstract] OR “rice porridge” [Title/Abstract] OR “hot porridge”
5#	1# OR 2#
6#	3# OR 4#
7#	5# AND 6#

### 2.5. Study selection and data extraction

#### 2.5.1. Study selection.

**Figure 1.**
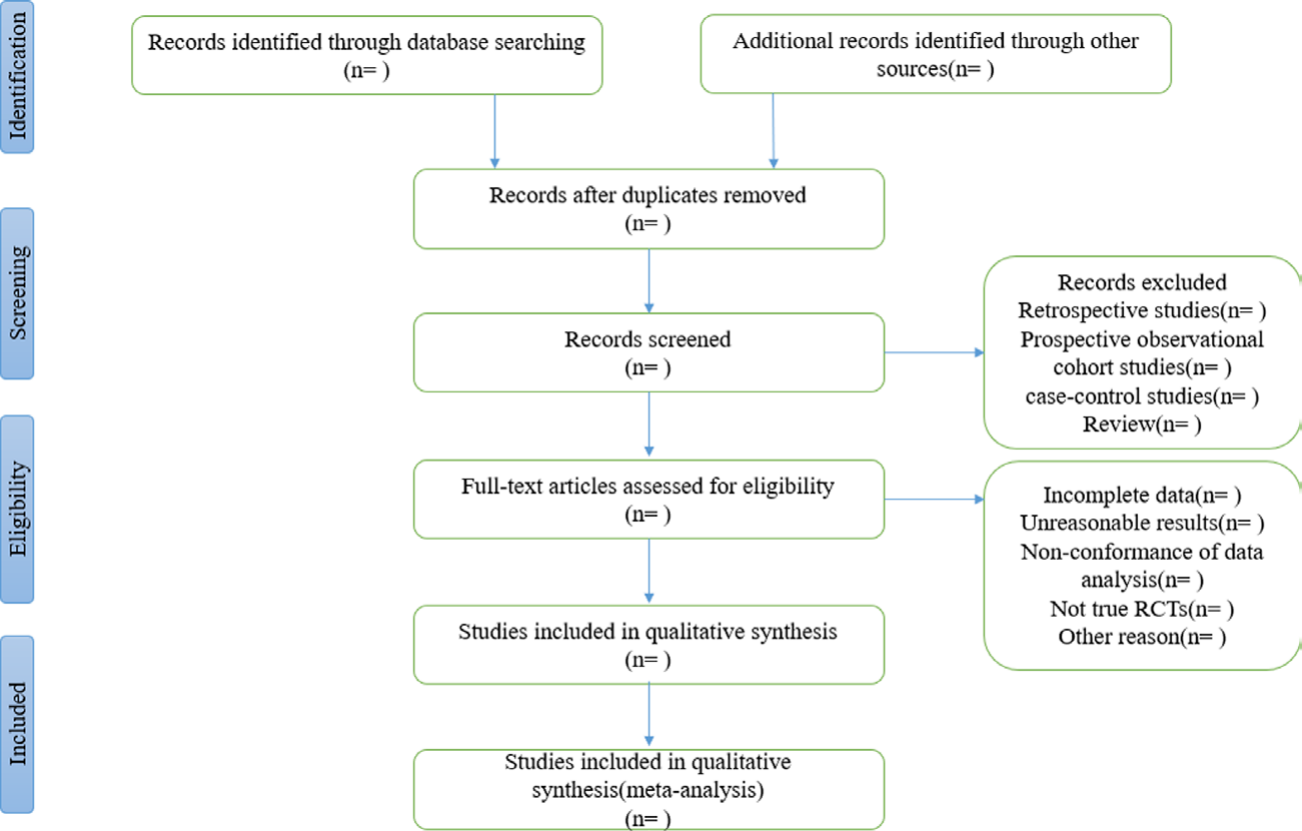
Flow chart and descriptions of study selection.

According to the predefined inclusion criteria, titles and abstracts of all records retrieved in the literature search will be scanned by 2 review authors. Trials that met the inclusion criteria will be retrieved for further assessment. All potentially relevant articles will be investigated via scrutinizing full text. The Systematic Reviews and Meta-Analyses flow diagram of study selection will be used to depict trial identification and exclusion process. Disagreements at study selection will be resolved by discussion and consensus. The detailed flowchart was displayed in Figure 1.

#### 2.5.2. Data extraction.

All eligible data will be extracted by 2 review authors and recorded according to standard data forms in duplicate. Extraction of all study data will include the following: trial characteristics (authors, country background, year of publication, study design, study duration), participant characteristics (mean age, gender, ethnicity), dose and frequency of use of porridge, and predefined primary and secondary results. All disagreements will be resolved through consultation or through a third reviewer. If the original data in the article is insufficient, the authors will be contacted by email for complete data.

### 2.6. Quality assessment of included studies

Two independent review authors will use Cochrane tool for assessing risk of bias to evaluate the methodological quality of included randomized controlled trials. The assessment details include 7 domains as follows: random sequence generation, allocation concealment, blinding of participants and personnel, blinding of outcome assessment, incomplete outcome data, selective reporting and other bias.^[16]^ We will resolve the differences in quality assessment through consultation or through third-party reviewers. For qualified research, each area will be defined as having a high, low, and ambiguous risk of bias.

### 2.7. Data synthesis and statistical analysis

Stata V.14.0 (StataCorp) will be used to carry out all statistical analyses. To assess the continuity of test results, we will use 95% CIs to aggregate mean differences. The overall estimates of the risk ratios and their associated 95% CIs will also be calculated for categorical outcomes. *P* < .05 will be regarded as statistically significant. The heterogeneous results included in the study will be established by a random-effects model. The heterogeneity across included trials will be assessed by *I*^2^ statistic. Heterogeneity will be considered to be low (*I*^2^ < 25%), moderate (*I*^2^ = 25%–50%) or high (*I*^2^ > 75%).^[17,18]^ Potential publication bias will be evaluated by funnel plots, and also assessed by the Egger and Begg tests if no <10 studies are pooled.

### 2.8. Sensitivity and subgroup analyses

In order to exclude studies with high bias risk, sensitivity analysis will be carried out. In order to ensure the stability of the evaluation results, the sensitivity analysis of retention samples will also be carried out. Moreover, outcome data on adults (18–64 years) and elderly population (aged 65 years or older) will be analyzed separately. Gender and ethnicity will also be considered for subgroup analyses if the data permit.

### 2.9. Assessment of publication bias

Egger test will be conducted to evaluate publication bias, and results displayed by funnel plot. If there is publication bias (*P* < .05), we would use a trim and fill method to adjust bias.

### 2.10. Quality assessment

The grading of recommendations assessment development and evaluation approach will be applied to rate the overall quality of evidence with respect to each outcome of our findings.^[19]^ The grading of recommendations assessment development and evaluation method categorizes the quality of evidence into 4 levels as follows: very low, low, moderate, and high. Reporting bias, research limitations, indirectness of evidence, inconsistency and inaccuracy of results may affect credibility.

## 3. Discussion

At present, the treatment of influenza is mainly based on antibiotics and antiviral drugs, but the abuse and resistance of antibiotics has become a very serious problem in China.^[20,21]^ Data show that 50% of outpatients in China use antibiotics. Among these outpatients, 74.0% used 1 antibiotic and 25.3% used 2 or more antibiotics.^[22,23]^ Porridge is not only a delicious food of the East, but also a good medicine for physicians of past dynasties to treat diseases. Physicians of past dynasties have recorded the use of porridge to treat influenza. Using porridge to treat influenza can reduce the abuse of antibiotics. In recent years, some doctors have suggested that they should eat more porridge during influenza, but others oppose the use of porridge for influenza, and there is some controversy about the use of porridge to treat influenza. Our study will be a systematic review and meta-analysis to evaluate the efficacy and safety of porridge in the treatment of influenza. The conclusion of this study has a certain reference value for the clinical use of porridge in the treatment of influenza.

## Acknowledgments

We appreciate the support of the fund and all authors who participated.

Provenance and peer review: Not commissioned; externally peer reviewed.

Patient consent for publication: Patient consent for publication.

## Author contributions

Sun H, Song J and Zhu W designed this study and Sun H was the main coordinators of this study. Mei M, Chen X and Xu J finished the initially searching of databases and provided search strategy of PubMed. Sun H and Mei M drafted this protocol. Sun H and Zhu W registered the protocol in PROSPERO. All authors have revised this protocol and approved its publication.

**Conceptualization:** Haoxiang Sun, Wei Zhu, Jianping Song.

**Investigation:** Jiayu Xu, Xiaofang Chen.

**Writing – original draft:** Haoxiang Sun, Manxue Mei.
